# High plasma adiponectin is associated with increased pulmonary blood flow and reduced right ventricular function in patients with pulmonary hypertension

**DOI:** 10.1186/s12890-020-01233-4

**Published:** 2020-07-30

**Authors:** Dongling Luo, Pengyuan Chen, Ziyang Yang, Yongheng Fu, Yigao Huang, Hezhi Li, Jimei Chen, Jian Zhuang, Caojin Zhang

**Affiliations:** 1 Department of Cardiology, Guangdong Provincial People’s Hospital, Guangdong Academy of Medical Sciences, Guangdong Cardiovascular Institute, Guangzhou, China; 2 Department of Cardiology, Guangdong General Hospital’s Nanhai Hospital, the Second Hospital of Nanhai District Foshan City, Foshan, China; 3 Department of Laboratory, Guangdong Provincial Key Laboratory of South China Structural Heart Disease, Guangdong Provincial People’s Hospital, Guangdong Academic of Medical Sciences, Guangzhou, China; 4 Department of Echocardiography, Guangdong Provincial People’s Hospital, Guangdong Academy of Medical Sciences, Guangdong Cardiovascular Institute, Guangzhou, China; 5 Department of Cardiac Surgery, Guangdong Provincial People’s Hospital, Guangdong Academy of Medical Sciences, Guangdong Cardiovascular Institute, Guangzhou, China

**Keywords:** Congenital heart disease, Pulmonary hypertension, N-terminal pro-brain natriuretic peptide, Adiponectin, Right ventricular function

## Abstract

**Background:**

Adiponectin is a biomarker closely related to heart failure. However, its role in pulmonary hypertension remains unclear. In this study, we investigated the association between adiponectin and hemodynamic abnormalities, right ventricular function in patients with congenital heart disease associated pulmonary hypertension (CHD-PH).

**Methods:**

Patients with CHD-PH were enrolled in this cross-sectional study. Linear regression analysis was performed to assess the association between adiponectin, N-terminal pro-Brain Natriuretic Peptide (NT-proBNP) and different clinical parameters. Results were depicted as beta-estimates(ß) with 95%-confidence intervals (95% CI). In addition, mediation and receiver operating characteristic curve analyses were used to analyze the relationships among adiponectin, NT-proBNP and right ventricular function.

**Results:**

A total of 86 CHD-PH patients were included. The overall mean adiponectin concentration was 7.9 ± 5.8 μg/ml. Log adiponectin was positively correlated with pulmonary circulation index (ß = 2.2, 95% CI 0.5, 4.0), log NT-proBNP (ß = 0.22, 95% CI 0.04, 0.41) and inversely with the tricuspid annular plane systolic excursion (TAPSE, ß = -4.7, 95% CI -8.6, − 0.8). The mediation analysis revealed the association between NT-proBNP and TAPSE was fully mediated by adiponectin (total effect c = − 5.4, 95% CI -9.4, − 1.5, *p* = 0.013; direct effect c’ = − 3.7, 95% CI -7.5, 0.1, *p* = 0.067). Additionally, the efficiency of adiponectin for detecting right ventricular dysfunction was not inferior to NT-proBNP (AUC = 0.84, 95% CI 0.67–1.00 vs AUC = 0.74, 95% CI 0.51–0.97, *p* = 0.23).

**Conclusions:**

Adiponectin is closely correlated with pulmonary blood flow and right ventricular function and may be a valuable biomarker for disease assessment in patients with pulmonary hypertension.

## Background

Pulmonary hypertension (PH) of variable degree is commonly accompanying with congenital heart disease (CHD) [1]. Closure on time can reverse, or even cure the disease. However, if not detected, the chronic exposure to high blood flow and pressure could trigger remodeling of the pulmonary vessels and finally leads to right heart failure [2]. Unfortunately, patients with PH are often asymptomatic until right ventricular dysfunction has developed, at which point irreversible remodeling may have occurred and the chance for shunt closure would have been lost [3].

Though plenty of biomarkers assessing the reversibility or disease severity have been reported in previous work, only N-terminal pro-Brain Natriuretic Peptide (NT-proBNP) correlates well with hemodynamic parameters and survival in PH patients and thus has been incorporated in clinical PH guidelines [4]. Recent studies suggest a positive association between adiponectin (APN) and NT-proBNP in heart failure patients [5, 6]. Furthermore, adiponectin has been found to be elevated in patients with pulmonary hypertension [7, 8] and positively correlated with increased blood flow and vascular resistance [9].

However, whether plasma adiponectin correlates with hemodynamics in patients with congenital heart disease associated pulmonary hypertension (CHD-PH) or is just as a confounder accompanying with increased NT-proBNP, has not been examined. Thus, in the present study we investigated the correlation between plasma adiponectin and hemodynamic abnormalities, right ventricular function in patients with CHD-PH and analyzed the potential relationship between NT-proBNP and adiponectin.

## Methods

### Study population

Eligible CHD patients were included in this cross-sectional study, from June 1st, 2016 to January 1st, 2017 at Guangdong Cardiovascular Institute. Patients were included if systemic-pulmonary shunting cardiac defects were reported in their echocardiogram and required further assessment by the right heart catheterization (RHC) after evaluated by their physicians. Patents who refused to receive the RHC, younger than 18 years old or with a mean pulmonary arterial pressure (mPAP) less than 25 mmHg, as confirmed by the RHC, were excluded. Besides, patients with history of Down’s syndrome, severe valvular heart disease, connective tissue disease or chronic thromboembolism disease, were also excluded in this study. The study protocol conformed to the ethical guidelines of the 1975 Declaration of Helsinki and was approval by the Ethics Committee of Guangdong General Hospital. Written informed consent was obtained from each patient.

### Data collection and adiponectin measurement

Demographic information, including age, gender, body mass index (BMI), systolic and diastolic blood pressure, were collected from the medical records. Blood samples were drawn from all participants at 6–8 am on the same day of the right heart catheterization (RHC) after an eight-hour overnight fasting. Biochemical measurements were performed in the laboratory of our institute. Biochemical parameters obtained for analysis included fasting blood glucose, serum creatinine, total cholesterol, triglyceride, high density lipoprotein (HDLC), low density lipoprotein cholesterol (LDLC) and NT-proBNP. NT-proBNP was quantitatively determined on the Roche Elecsys 2010 immunoassay analyzer, using electrochemiluminescence immunoassay technique in our laboratory.

For adiponectin measurement, 2 ml blood was drawn during the process of RHC and collected in plastic tubes to which disodium ehtylenediamine tetraacetic acid (EDTA) were added. The tubes were immediately taken to the laboratory, centrifuged at degree centigrade for separation of the plasma, and the plasma was then frozen and stored at − 80 degree centigrade until analysis. Serum adiponectin levels were measured using a commercially available monoclonal and recombinant human adiponectin (R&D systems, Abingdon, UK). The intra-assay coefficient of variation (CV) was < 5% and the inter-assay CV was < 10%.

### Echocardiograph

Echocardiograph was performed one day to one week before RHC using a commercially available probe and system (PHILIPS IE33). The echocardiographic indices, including left atrium dimension (LA), left ventricular end-diastolic dimension (LVEDD), left ventricular end-systolic dimension (LVESD), right atrium dimension (RA) and right ventricular end-diastolic diameter (RVEDD) at the midlevel, were measured according to the guidelines of the American Society of Echocardiography [10]. The left ventricular ejection fraction (LVEF) was measured using Simpson’s biplane method. Tricuspid annular plane systolic excursion (TAPSE) and systolic velocity of lateral tricuspid annulus displacement (S′) were measured to represent for the right ventricular function. In the present study, either TAPSE < 16 mm or S′ < 10 cm/s was considered as reduced right ventricular function or right ventricular dysfunction [11]. Due to the large quantity of patients in China, indexes for right heart ventricular function, like TAPSE, S′, were not routinely assessed and only evaluated in patients with enlarged right heart or potential risk of right ventricular dysfunction. Therefore, missing data for right ventricular function was inevitable in this study and thus we could only perform subgroup analysis for this part.

### Right heart catheterization

The right heart catheterization was performed by two experienced experts in our institute. The right atrium pressure (RAP), pulmonary artery pressure (PAP) and pulmonary artery wedge pressure (PAWP) were measured via the 6-French MP catheter. Blood samples were aspirated from superior vena cava, inferior vena cava, pulmonary artery and left heart system. Oxygen saturation of the blood sample was determined by using a standard blood gas analyzer (GEM Premier 3000). Subsequently, oxygen contents of the arterial and venous blood were calculated to determine the oxygen consumption (VO2). In the Fick method [12], the systemic (cardiac output, CO), pulmonary blood flow (pulmonary output, PO) and the corresponding cardiac index (CI), pulmonary index (PI) were estimated by the following equations:

- Systemic blood flow (CO, L/min) = VO2 (ml/min) / (systemic arterial saturation- mixed venous oxygen saturation) *1.34 Hemoglobin(g/L) *10
- Pulmonary blood flow (PO, L/min) = VO2 (ml/min) / (pulmonary venous oxygen saturation-mixed venous oxygen saturation) *1.34 Hemoglobin(g/L) *10
- Cardiac index (CI, L/min/m^2^) = CO(L/min) / Body surface area (m^2^)
- Pulmonary index (PI, L/min/m^2^) = PO(L/min) / Body surface area (m^2^)

Pulmonary vascular resistance (PVR) was calculated by mean pulmonary arterial pressure divided by the cardiac output.

### Statistical methods

Results were expressed as mean ± standard deviation for continuous variables or as number of patients and percentages for categorical variables. Differences between continuous variables were assessed using student’s t-test. Categorical variables were compared among groups by the chi-square test or Fisher’s test, as appropriate. Because serum levels of adiponectin and NT-proBNP were not normally distributed, log 10 scale transformation of adiponectin and NT-proBNP (log adiponectin and log NT-proBNP) were used in this study. Correlation between log adiponectin and log NT-proBNP was assessed by using the Pearson’s correlation analysis. Subsequently, linear regression models were used to estimate the non-adjusted or adjusted (age, gender, BMI, creatinine, TRIG/HDLC and glucose levels) association between adiponectin, NT-proBNP and echocardiographic, hemodynamic parameters.

Mediation analysis was performed to assess the potential mechanistic relationship between NT-proBNP and adiponectin on right ventricular function. In the mediation model, linear regression analysis was performed. a is the coefficient relating the independent variable to the mediator, b is the coefficient relating the mediator to the dependent variable adjusted for the independent variable, c’ is the coefficient relating the independent variable to the dependent variable adjusted for the mediator [13]. In order to eliminate the potential effects of confounders, this model was adjusted by age, gender, BMI, creatinine, TRIG/HDLC and glucose level.

Receiver operating characteristic (ROC) curve analyses were performed and the area under the curve (AUC) was calculated to evaluate the diagnostic efficiency of APN and NT-proBNP for right ventricular dysfunction. The Z-test was also performed to determine the statistical difference of the ROC curve. All *p*-values were 2-sided, and a value at *p* < 0.05 was considered statistically significant. All analyses were performed using Empower(R) (www.empowerstats.com, X&Y solutions, inc. Boston MA) and R (http://www.R-project.org).

## Results

Of the 203 eligible patients, 117 had a mean pulmonary arterial pressure less than 25 mmHg and were excluded. The mean age of the study population was 35.8 ± 13.2 years old and 68% (59/86) were female. The overall mean plasma APN level was 7.9 ± 5.8 μg/ml and NT-proBNP was 468.6 ± 1011.5 pg/ml. Among the included 86 CHD-PH participants, 52 were caused by atrial septal defect (ASD), 21 by ventricular septal defect (VSD), 9 by patent ductus arteriosus (PDA) and 4 by coexisting of PDA and VSD or ASD. 58% (50/86) of them were in the class of NYHA I-II while the remaining 42% were in NYHA III-IV. Finally, for the included patients, 28 (32.5%) received shunt closure by percutaneous intervention, 25(29.0%) received open heart surgery and 30 (34.9%) started on targeted therapy (either monotherapy or combined therapy of endothelin antagonist, prostanoids, phosphodiesterase 5 inhibitor). Three patients refused to receive surgical managements and were closely followed up after discharged. Other demographic and clinical data are presented in Table 1.

**Table 1:** Baseline characteristic of the included patients

	Valid cases, n(%)	Values
**Clinical characteristics**		
Age, years	86 (100)	35.8 ± 13.2
Gender, female	86 (100)	59 (68%)
BMI, kg/m2	86 (100)	21.2 ± 4.0
**Laboratory measurements**		
Hemoglobin, g/l	86 (100)	139.2 ± 22.9
Adiponectin, ug/ml	86 (100)	7.9 ± 5.8
NT-proBNP, pg/ml	72 (84)	468.6 ± 1011.5
Glucose, mmol/l	86 (100)	4.9 ± 1.4
Creatinine, umol/l	86 (100)	64.2 ± 15.1
CHOL, mmol/l	81 (94)	4.4 ± 0.8
TRIG, mmol/l	81 (94)	1.2 ± 0.9
HDLC, mmol/l	81 (94)	1.2 ± 0.2
LDLC, mmol/l	81 (94)	2.8 ± 0.7
TRIG/HDLC	81 (94)	1.1 ± 1.2
**Echocardiography**		
LVEF,%	86 (100)	63.6 ± 9.9
LA, mm	86 (100)	35.9 ± 7.7
LVEDD, mm	86 (100)	44.3 ± 10.1
LVESD, mm	86 (100)	27.9 ± 8.3
RA, mm	86 (100)	56.8 ± 15.8
RVEDD, mm	86 (100)	60.4 ± 10.4
TAPSE, mm	41 (48)	21.0 ± 5.4
S′, cm/s	41 (48)	13.4 ± 3.9
**Right heart catheterization**		
mRAP, mmHg	86 (100)	5.8 ± 2.9
mPAP, mmHg	86 (100)	41.7 ± 23.7
PAWP, mmHg	86 (100)	9.7 ± 2.9
MOS,%	86 (100)	70.0 ± 10.5
PVR, wood units	86 (100)	5.7 ± 5.9
PI, L/min/m2	86 (100)	6.8 ± 3.3
CI, L/min/m2	86 (100)	3.7 ± 1.0
QP/QS	86 (100)	1.9 ± 0.9
RP/RS	86 (100)	0.3 ± 0.3

*Abbreviation*: *BMI* body mass index, *CHOL* cholesterol, *TRIG* triglyceride, *HDLC* high density lipoprotein cholesterol, *LDLC* high density lipoprotein cholesterol, *TRIG/HDLC* the ratio of TRIG and HDLC, *LVEF* left ventricular ejection fraction, *LA* left atrium, *LVEDD* left ventricular end-diastolic dimension, *LEVSD* left ventricular end-systolic dimension, *RA* right atrium, *RVEDD* right ventricle end-diastolic dimension, *TAPSE* tricuspid annular plane systolic excursion, *S′* systolic velocity of lateral tricuspid annulus displacement, *RVFAC* right ventricular functional area change, *mRAP* mean right atrial pressure, *mPAP* mean pulmonary arterial pressure, *MOS* mixed venous oxygen saturation, *PVR* pulmonary vascular resistance, *PI* pulmonary circulation index, *CI* cardiac index, *QP/QS* the ratio of pulmonary circulation and systemic circulation blood flow, *RP/RS* the ratio of pulmonary vascular resistance and systemic vascular resistance

### Association between APN and different clinical parameters

**Fig. 1:**
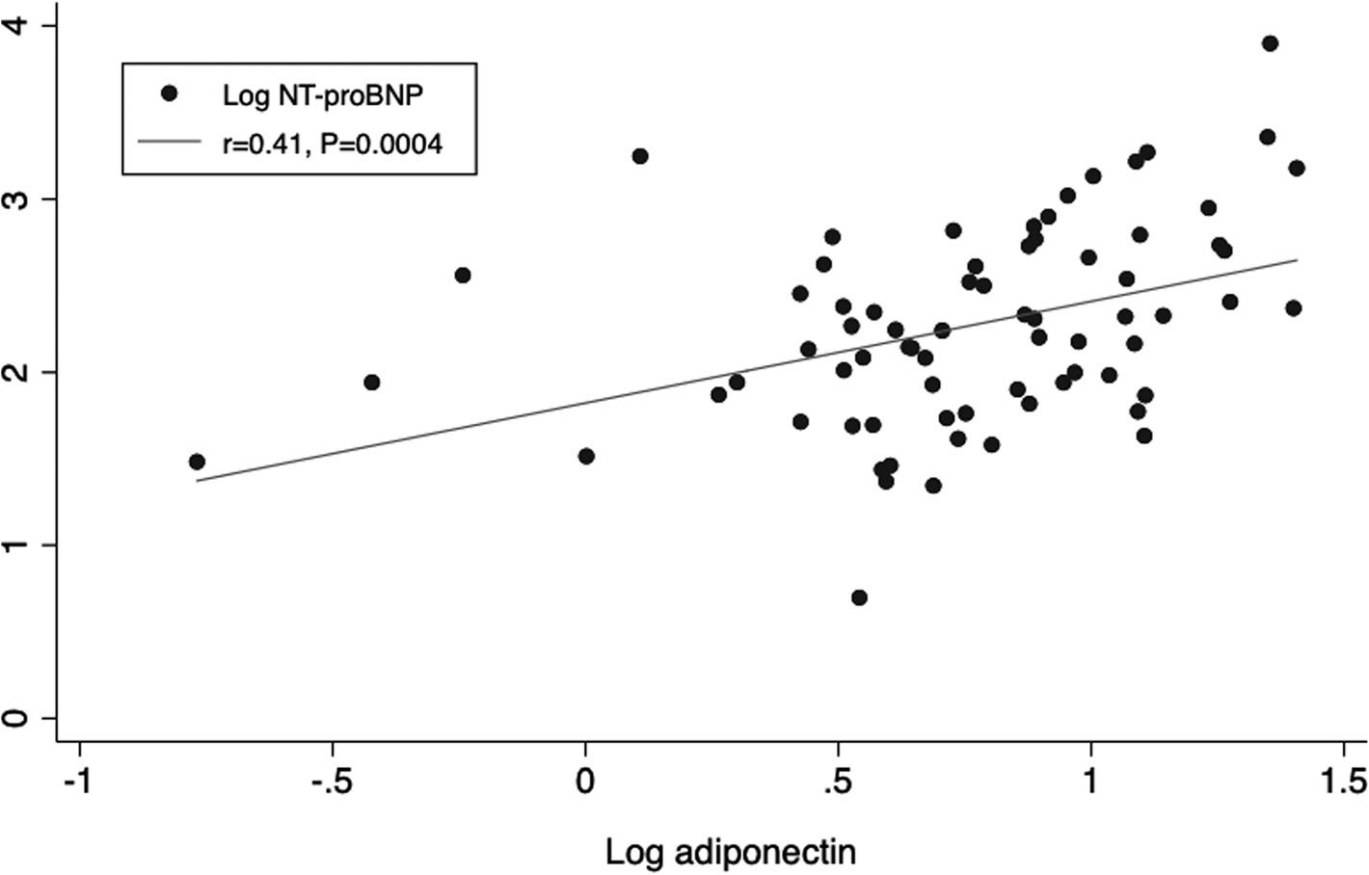
Correlation between log adiponectin and log NT-proBNP. Log adiponectin is positively correlated with log NT-proBNP (r = 0.41, *p* = 0.0004). Log adiponectin and log NT-proBNP are log 10 scale transformation of plasma levels of adiponectin and NT-proBNP

On the Pearson’s correlation analyses, the result revealed that log adiponectin was positively correlated with log NT-proBNP (r = 0.41, *p* < 0.001, Fig. 1). Table 2 presents the linear regression analyses between log adiponectin and different clinical parameters. There were no statistically significant correlations between log adiponectin and pulmonary vascular resistance, cardiac index, mixed venous oxygen saturation, mean right atrial pressure or the cardiac structure of the left side. Interestingly, log adiponectin levels were positively correlated with pulmonary circulation index, even after adjustment for age, gender, BMI, creatinine, TRIG/HDLC and glucose levels. In addition, log adiponectin levels correlated positively with RVEDD but negatively with TAPSE and S′.

**Table 2:** Linear regression analysis of adiponectin, NT-proBNP and different clinical parameters

	Log Adiponectin		Log NT-proBNP	
	Non-adjusted	Adjusted	Non-adjusted	Adjusted
mRAP	1.2 (− 0.4, 2.8) 0.133	0.1 (−1.5, 1.7) 0.903	2.2 (1.0, 3.3) < 0.001	1.7 (0.3, 3.0) 0.020
mPAP	7.7 (− 5.2, 20.6) 0.248	3.8 (− 10.5, 18.1) 0.601	15.8 (6.9, 24.7) < 0.001	24.7 (14.6, 34.8) < 0.001
MOS	3.5 (− 2.2, 9.2) 0.232	5.5 (− 0.9, 11.9) 0.096	−3.9 (−8.3, 0.6) 0.096	−5.3 (− 11.0, 0.5) 0.077
PVR	0.6 (− 2.7, 3.8) 0.731	− 1.1 (− 4.0, 1.8) 0.471	2.1 (− 0.3, 4.5) 0.092	2.7 (0.4, 4.9) 0.023
PI	2.2 (0.5, 4.0) 0.013	2.1 (0.2, 4.1) 0.036	1.0 (−0.3, 2.3) 0.150	0.7 (− 1.0, 2.4) 0.445
CI	0.1 (−0.5, 0.6) 0.862	0.2 (−0.4, 0.9) 0.477	− 0.6 (− 1.0, − 0.2) 0.005	−0.7 (− 1.3, − 0.2) 0.013
LA	4.3 (− 0.0, 8.6) 0.057	4.2 (− 0.6, 9.0) 0.089	3.5 (0.5, 6.5) 0.026	4.2 (0.2, 8.2) 0.048
LVEDD	2.4 (− 3.3, 8.2) 0.409	3.5 (− 3.2, 10.2) 0.311	1.2 (− 3.6, 6.0) 0.624	4.5 (− 2.0, 11.0) 0.181
LVESD	3.6 (− 1.1, 8.3) 0.139	3.8 (− 1.5, 9.0) 0.164	2.7 (− 1.1, 6.5) 0.176	4.7 (− 0.3, 9.7) 0.071
LVEF	−8.5 (− 13.9, − 3.1) 0.003	− 8.3 (− 13.6, − 2.9) 0.004	−6.0 (− 10.4, − 1.6) 0.010	−5.8 (− 11.1, − 0.5) 0.038
RA	10.4 (1.6, 19.2) 0.023	7.5 (− 2.0, 16.9) 0.126	10.7 (6.0, 15.4) < 0.001	6.1 (0.4, 11.8) 0.043
RVEDD	9.5 (3.9, 15.0) 0.001	7.2 (1.2, 13.2) 0.022	10.2 (6.5, 14.0) < 0.001	10.4 (5.8, 14.9) < 0.001
TAPSE	−4.7 (− 8.6, − 0.8) 0.022	− 4.9 (− 9.2, − 0.5) 0.036	−5.2 (− 7.8, − 2.5) < 0.001	−5.4 (− 9.4, − 1.5) 0.013
S‘	−3.0 (− 5.9, − 0.2) 0.042	−3.7 (− 6.7, − 0.7) 0.024	−2.2 (− 4.3, 0.0) 0.059	−2.0 (− 4.8, 0.9) 0.189

*Abbreviation*: *mRAP* mean right atrial pressure, *mPAP* mean pulmonary arterial pressure, *MOS* mixed venous oxygen saturation, *PVR* pulmonary vascular resistance, *PI* pulmonary circulation index, *CI* cardiac index, *LVEF* left ventricular ejection fraction, *LA* left atrium, *LVEDD* left ventricular end diastolic dimension, *LEVSD* left ventricular end-systolic dimension, *RA* right atrium, *RVEDD* right ventricle end-diastolic dimension, *TAPSE* tricuspid annular plane systolic excursion, *S′* systolic velocity of lateral tricuspid annulus displacement

Adjusted, adjusted by age, gender, BMI, creatinine, TRIG/HDLC and glucose levels

Depicted are beta-estimates with 95%-confidence intervals and *p*-value from linear regression analysis

### Association between NT-proBNP and different clinical parameters

Also depicted in Table 2, log NT-proBNP was shown to be positively correlated with mean right atrial pressure, mean pulmonary artery pressure, dimension of the left atrium and right atrium, right ventricle end-diastolic dimension and negatively correlated with cardiac index, left ventricular ejection fraction and TAPSE, either in adjusted or non-adjusted models. Though the association of log NT-proBNP and pulmonary vascular resistance was not statistically significant in the non-adjusted model, a positive correlation was observed after adjusting for age, gender, BMI, creatinine, TRIG/HDLC and glucose level. Of note, no significant correlation was observed between log NT-proBNP and pulmonary circulation index.

### Association between NT-proBNP, APN and right ventricular function

**Fig. 2:**
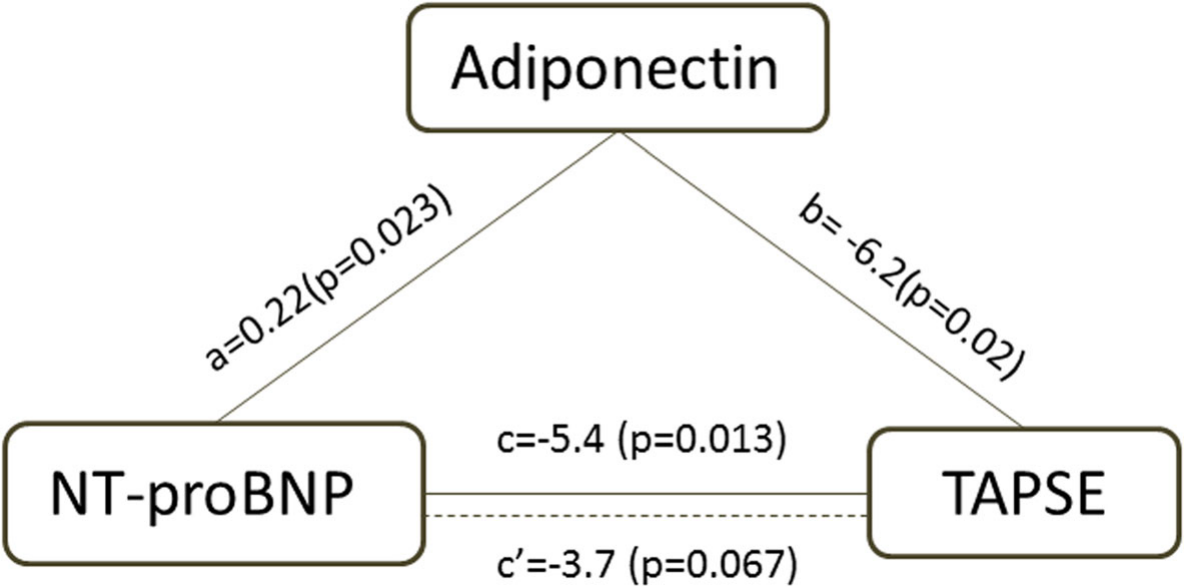
The mediation effect of adiponectin (APN) on the association between NT-proBNP and right ventricular dysfunction. a is the coefficient between log NT-ProBNP and log adiponectin (a = 0.22, 95%CI 0.04, 0.41, *p* = 0.023). b is the coefficient between log adiponectin and TAPSE (b = − 6.2, 95%CI -11.0, − 1.4, p = 0.02). c is the coefficient between log NT-proBNP and TAPSE (c = − 5.4, 95%CI -9.4, − 1.5, *p* = 0.013). The coefficient c’ between log NT-proBNP and TAPSE becomes insignificant when log adiponectin is included in the model (c’ = − 3.7, 95%CI -7.5, 0.1, *p* = 0.067). These results reveal that the association between NT-proBNP and right ventricular dysfunction, as assessed by TAPSE, is fully mediated by APN. TAPSE, tricuspid annular plane systolic excursion

**Fig. 3:**
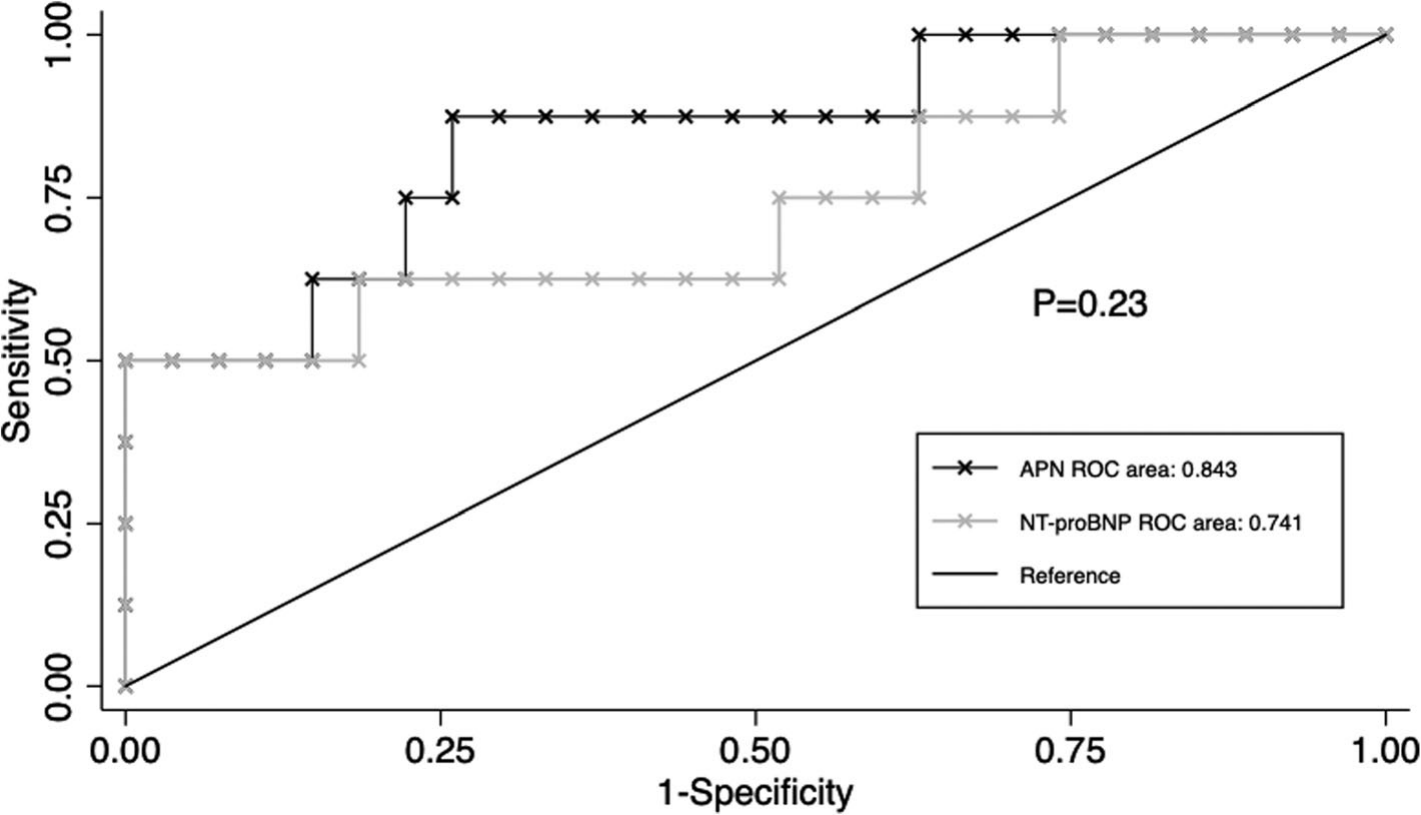
Receiver operating characteristic curve of adiponectin and NT-proBNP. There’s a trend that adiponectin exhibited a better diagnostic efficiency as compared to NT-proBNP in patients with right ventricular dysfunction (AUC = 0.84, 95%CI 0.67–1.00; AUC = 0.74, 95%CI 0.51–0.97, respectively, *p* = 0.23). AUC, area under the curve

As mentioned previously, log adiponectin was positively correlated with log NT-proBNP. Since both log NT-proBNP and log adiponectin were associated with TAPSE, a mediation analysis was performed. As illustrated in Fig. 2, the regression coefficients between log NT-proBNP and log adiponectin (a = 0.22, 95% CI 0.04, 0.41, *p* = 0.023), log adiponectin and TAPSE (b = − 6.2,95% CI -11.0, − 1.4, p = 0.02) were both significant. The total effect of log NT-proBNP on TAPSE became smaller when potential mediator log APN was included in the model (total effect c = − 5.4, 95% CI -9.4, − 1.5, *p* = 0.013; direct effect c’ = − 3.7, 95% CI -7.5, 0.1, *p* = 0.067). These results revealed that the association between NT-proBNP and right ventricular dysfunction, as assessed by TAPSE, was fully mediated by APN. In addition, as depicted in Fig. 3, the diagnostic efficiency of adiponectin for detecting right ventricular dysfunction was not inferior or even probably superior to NT-proBNP (AUC = 0.84, 95% CI 0.67–1.00; AUC = 0.74, 95% CI 0.51–0.97, respectively, *p* = 0.23).

## Discussion

In the present study, adiponectin levels were found to be positively correlated with the pulmonary circulation blood flow and negatively with the right ventricular function in patients with CHD-PH. In addition, when compared with NT-proBNP, an at least non-inferior diagnostic efficiency for right ventricular dysfunction was observed for adiponectin and further, the association of NT-proBNP and right ventricular dysfunction was probably mediated by the effect of adiponectin.

### Adiponectin and CHD-PH

Patients with CHD-PH had significantly higher serum adiponectin (7.9 ± 5.8 μg/ml) as compared to a group of healthy adults from our hospital (3.61 ± 2.87 μg/ml) and a healthy control group of a Korean study (5.99 ± 2.75 μg/ml), with similar age, BMI and sex distribution [14]. This suggests a role of adiponectin in patients with CHD-PH. Adiponectin is an adipokine, which was known to be almost exclusively produced and secreted by adipocytes. However, in addition to adipocytes, various other cell types secrete adiponectin [15]. Recent studies have reported adiponectin expression in cardiomyocytes and endothelial cells [16–18]. It’s been reported that adiponectin could reduce accumulation of inflammatory cells, inhibit proliferation of vascular smooth muscle cells and thus exerts a cardio-protective effect in pulmonary hypertension [19, 20]. However, emerging clinical observations demonstrate that plasma APN levels are elevated in patients with PH and positive correlated with pulmonary vascular resistance (PVR) and pulmonary arterial systolic pressure (PASP) [8]. Furthermore, the elevated adiponectin is associated with poorer cardiac function and higher mortality in heart failure patients [21, 22]. Though we did not find significant correlation between adiponectin and PVR as well as the pulmonary arterial pressure, we first demonstrated that adiponectin was closely related to pulmonary circulation blood flow. As suggested in previous studies, increased pulmonary blood flow is regarded the essential trigger for PH development and should closely be monitored [23, 24]. Additionally, adiponectin was positively associated with right ventricular dimension and negatively with right ventricular function, which suggested APN might be an important indicator for the pulmonary-right ventricular system.

These findings were in line with the previous studies [11, 25, 26]. The level of adiponectin was higher in patients with right ventricular dysfunction and inversely correlated with TAPSE [8] or even an early RV dysfunction indicator, the right ventricular free wall global strain [27]. Furthermore, the concentration of APN correlated well with the change of RV function. Also, in the studies conducted by Serrano-Ferrer. et al. and Isobe S. et al., adiponectin levels reduced after improvement of right heart overload and restoration of the RV function [8, 27]. Besides, high plasma adiponectin was associated with a lower peak VO_2_ and higher VE/ VCO_2_-slope, parameters closely related to PH mortality [3, 26]. All these results underlined the importance of plasma adiponectin in assessing the right ventricular function in patients with CHD-PH.

However, the reasons for these observations remain to be unraveled. Some authors suggest that adiponectin plays a permissive role in the structural and metabolic cardiac remodeling and thus accelerates the transition to heart failure [28]. While others state that the association of hyperadiponectinemia and increased mortality in heart failure was a phenomenon of ‘adiponectin resistance’, characterized by downregulation of AdipoR1 and phosphorylation of the downstream proteins like AMPK and P38MAPK [29]. However, it is unclear why this occurs and needs further elucidation.

### Adiponectin and NT-proBNP

NT-proBNP is a well-recognized biomarker that has been recommended in the existing PH guidelines [4]. Consistent with previous studies, our study also demonstrated a positive correlation between NT-proBNP and PVR as well as mPAP. In addition, NT-proBNP positively correlated with adiponectin and negatively with the left and right ventricular function, as assessed by LVEF and TAPSE. However, unlike adiponectin, no significant relation of NT-proBNP to pulmonary blood flow was observed in the current study.

Recently, the association between NT-proBNP and adiponectin has been examined in adults with heart failure [5, 30, 31]. Some studies stated that the association of APN and heart failure vanished after adjusting for NT-proBNP [31], some revealed that high APN level was associated with increased risk of mortality, independent of plasma NT-proBNP [22], while Dai Z et al. found an improved diagnostic value of conjunction of NT-proBNP and APN for heart failure [30]. While in our study, the diagnostic efficiency of adiponectin for detecting right ventricular dysfunction was not inferior or even probably better than NT-proBNP. With respect to a possible mechanism accounting for these observations, studies are going on. As reported by Tsukamoto et al., increased adiponectin gene expression and adiponectin secretion were observed in cultured human adipocytes in response to natriuretic peptide treatment [32]. However, some other studies revealed that adiponectin was present in damaged cardiomyocytes and might be directly synthesized by and released from the failing heart [16, 17, 25].

Although whether the elevation of adiponectin is the result of increased NT-proBNP or as an active contributor to worsening heart failure progression remains unclear, some findings suggested that the effects of NT-proBNP might relate to adiponectin signaling [33]. As demonstrated by Masuch A et al., the effect of NT-proBNP on lipid prolife was partially mediated by adiponectin [33]. By using the mediation effect model, we also found a mediation effect of adiponectin on the association of NT-proBNP and right ventricular function. From this perspective of view, it is important to more fully investigate the effects of adiponectin and NT-proBNP on cardiac dysfunction based on basic experiments.

### Limitation

Due to the retrospective nature of this study, no causality of the interrelations between these parameters can be determined. In addition, limited by the small sample size in a single-center study, generalization of these findings to other types of PH necessitates further validation. Although TAPSE is a widely used index for right ventricular systolic performance, it’s only partially representative of global RV function and not as accurate and precise as that assessed by cardiac magnetic resonance [11]. What’s more, half of our patients had missing data for the echocardiographic assessment of the right ventricular function. However, patients with missing data had similar hemodynamic information, lower adiponectin level and less severe change of the right side of the heart (data depicted in Additional file 1), which indicated less right ventricular dysfunction and a higher specificity would be observed if all of them were included in the ROC analysis. Lastly, adiponectin has multiple isoforms (low, medium and high molecular weight (HMW)), with the HMW isoform being the most abundant and presenting the greatest biological properties [6, 34–36]. However, we did not evaluate the oligomeric state of the adiponectin. Although in a report of adiponectin and left ventricular function, total circulating adiponectin reflects well with the HMW adiponectin on the heart [37], we could not confirm this result in the present study owing to insufficient data. Therefore, further studies must be performed to better understand the role of different oligomeric state in patients with pulmonary hypertension and to clarify whether pulmonary hypertension causes alterations in expression or profile of adiponectin.

## Conclusion

Unlike other types of PH, earlier detection of pulmonary arteriopathy or right ventricular dysfunction could provide timely shunt closure and thus largely improve the survival of patients with CHD-PH. From the current findings of this study, adiponectin was closely correlated with pulmonary blood flow and right ventricular function, which might be a valuable biomarker for assessing the hemodynamics and prognosis in CHD patients with PH. However, knowledge gaps remain and further investigation is warranted.

## Supplementary information

**Additional file 1: Table S1.** Baseline characteristics in patients with (RVF group) or without (missing group) data of right ventricular function(RVF).

## Data Availability

The datasets used and/or analyzed during the current study are available from the corresponding author on reasonable request.

## Abbreviations

CHD-PH: congenital heart disease associated pulmonary hypertension
NT-proBNP: N-terminal pro-Brain Natriuretic Peptide
TAPSE: Tricuspid annular plane systolic excursion
PH: Pulmonary hypertension
CHD: Congenital heart disease
APN: Adiponectin
RHC: Right heart catheterization
mPAP: Mean pulmonary arterial pressure
BMI: Body mass index
HDLC: High density lipoprotein
LDLC: Low density lipoprotein cholesterol
TRIG/HDLC: the ratio of triglyceride and high density lipoprotein
LA: Left atrium dimension
LVEDD: Left ventricular end-diastolic dimension
LVESD: Left ventricular end-systolic dimension
RA: Right atrium dimension
RVEDD: Right ventricular end-diastolic diameter
LVEF: Left ventricular ejection fraction
S′: Systolic velocity of lateral tricuspid annulus displacement
RAP: Right atrium pressure
PAWP: Pulmonary artery wedge pressure
VO2: Oxygen consumption
CO: Cardiac output
PO: Pulmonary output
CI: Cardiac index
PI: Pulmonary index
PVR: Pulmonary vascular resistance
ROC: Receiver operating characteristic
AUC: Area under the curve
ASD: Atrial septal defect
VSD: Ventricular septal defect
PDA: Patent ductus arteriosus
RV: Right ventricle
NYHA: New York Heart Association
PASP: Pulmonary arterial systolic pressure
AMPK: Adenosine 5′-monophosphate-activated protein kinase
P38MAPK: P38 mitogen-activated protein kinase
HMW: High molecular weight
