# Supplementary material for: High plasma adiponectin is associated with increased pulmonary blood flow and reduced right ventricular function in patients with pulmonary hypertension
Source: BMC Pulm Med. 2020 Jul 30;20:204. doi: 10.1186/s12890-020-01233-4 (PMC7391602; doi:10.1186/s12890-020-01233-4)
Supplement: Supplementary file 1 — Additional file 1: Table S1. Baseline characteristics in patients with (RVF group) or without (missing group) data of right ventricular function(RVF). [file 12890_2020_1233_MOESM1_ESM.docx]

Supplementary table 1 Baseline characteristics in patients with (RVF group) or without (missing group) data of right ventricular function(RVF)

|  | Missing group(n=46) | RVF group(n=41) | P-value |
| --- | --- | --- | --- |
| **Clinical characteristics** |  |  |  |
| Age, years | 34.2 ± 13.8 | 37.5 ± 12.4 | 0.24 |
| Gender, female | 33 (71.7%) | 26 (63.4%) | 0.41 |
| BMI, kg/m2 | 21.1 ± 4.5 | 21.4 ± 3.3 | 0.72 |
| **Laboratory measurements** |  |  |  |
| Hemoglobin, g/l | 136.1 ± 22.6 | 142.6 ± 23.0 | 0.19 |
| Adiponectin, ug/ml | 6.9 ± 5.1 | 8.9 ± 6.5 | 0.12 |
| NT-proBNP, pg/ml | 337.0 ± 481.6 | 607.8 ± 1360.9 | 0.26 |
| Glucose, mmol/l | 4.6 ± 1.1 | 5.1 ± 1.7 | 0.13 |
| Creatinine, umol/l | 61.8 ± 15.2 | 66.9 ± 14.6 | 0.11 |
| CHOL, mmol/l | 4.4 ± 0.9 | 4.4 ± 0.7 | 0.94 |
| TRIG, mmol/l | 1.0 ± 0.4 | 1.4 ± 1.2 | 0.007 |
| HDLC, mmol/l | 1.2 ± 0.2 | 1.1 ± 0.2 | 0.10 |
| LDLC, mmol/l | 2.8 ± 0.7 | 2.8 ± 0.6 | 0.95 |
| TRIG/HDLC | 0.8 ± 0.4 | 1.4 ± 1.8 | 0.04 |
| **Echocardiography** |  |  |  |
| LVEF,% | 65.1 ± 9.7 | 62.4 ± 10.1 | 0.28 |
| LA, mm | 36.4 ± 8.3 | 35.5 ± 7.2 | 0.65 |
| LVEDD, mm | 47.5 ± 12.1 | 41.8 ± 7.3 | 0.02 |
| LVESD, mm | 29.6 ± 9.8 | 26.5 ± 6.7 | 0.14 |
| RA, mm | 50.3 ± 9.4 | 62.1 ± 18.0 | 0.002 |
| RVEDD, mm | 55.9 ± 10.9 | 64.1 ± 8.4 | <0.001 |
| **Right heart catheterization** |  |  |  |
| mRAP, mmHg | 5.4 ± 2.6 | 6.2 ± 3.2 | 0.19 |
| mPAP, mmHg | 39.4 ± 22.5 | 44.3 ± 25.0 | 0.34 |
| PAWP, mmHg | 10.1 ± 2.9 | 9.3 ± 2.9 | 0.22 |
| MOS,% | 71.3 ± 6.8 | 68.5 ± 13.4 | 0.21 |
| PVR, wood units | 4.7 ± 3.6 | 6.7 ± 7.6 | 0.11 |
| PI, L/min/m2 | 6.5 ± 2.8 | 7.0 ± 3.8 | 0.47 |
| CI, L/min/m2 | 3.8 ± 0.9 | 3.5 ± 1.2 | 0.19 |
| QP/QS | 1.7 ± 0.8 | 2.0 ± 0.9 | 0.11 |
| RP/RS | 0.3 ± 0.2 | 0.4 ± 0.4 | 0.28 |

Abbreviation: BMI, body mass index; CHOL, cholesterol; TRIG, triglyceride; HDLC, high density lipoprotein cholesterol; LDLC, high density lipoprotein cholesterol; TRIG/HDLC, the ratio of TRIG and HDLC; LVEF, left ventricular ejection fraction; LA, left atrium; LVEDD, left ventricular end-diastolic dimension; LEVSD, left ventricular end-systolic dimension; RA, right atrium; RVEDD, right ventricle end-diastolic dimension; TAPSE, tricuspid annular plane systolic excursion; S’, systolic velocity of lateral tricuspid annulus displacement ; RVFAC, right ventricular functional area change; mRAP, mean right atrial pressure; mPAP, mean pulmonary arterial pressure; MOS, mixed venous oxygen saturation; PVR, pulmonary vascular resistance; PI, pulmonary circulation index; CI, cardiac index; QP/QS, the ratio of pulmonary circulation and systemic circulation blood flow; RP/RS, the ratio of pulmonary vascular resistance and systemic vascular resistance.
